# Bayesian inference of plasma parameters from collective Thomson scattering technique on a gas-puff near stagnation

**DOI:** 10.1038/s41598-023-40014-x

**Published:** 2023-08-10

**Authors:** M. Escalona, J. C. Valenzuela, G. Avaria, F. Veloso, E. S. Wyndham

**Affiliations:** 1 Instituto de Física, Pontificia Universidad Católica de Chile, Av. Vicuña Mackenna 4860, Macul, Chile; 2 Research Center on the Intersection in Plasma Physics, Matter and Complexity, P2mc, Comisión Chilena de Energía Nuclear, Casilla 188-D, Santiago, Chile

**Keywords:** Laser-produced plasmas, Magnetically confined plasmas, Characterization and analytical techniques

## Abstract

The Collective Thomson scattering technique has been implemented to study the stagnation of a single liner gas-puff. The plasma parameters are determined by theoretically modelling the scattering form factor in combination with Bayesian inference to provide the set of the most probable parameters that describe the experimental data. Analysis of the data reveal that incoming flows are able to interpenetrate partially. Estimation of the mean free path shows a gradual transition from a weakly collisional to a collisional regime as the plasma gets to the axis. Furthermore, we find that the ion energy at $\mathrm{r}=2.5\,\mathrm{mm}$ is ${13.6}_{-0.9}^{+1.0}\,\mathrm{keV}$ and is mostly kinetic in nature and represents ${98}_{-9}^{+10} \%$ of the total energy. This kinetic energy is far greater than the value on axis of ${3.7}_{-0.5}^{+0.4}\,\mathrm{keV}$ which is ${84}_{-14}^{+15} \%$ of the total energy. Energy transfer to the electrons and radiation losses are found to be negligible by this time. A possible explanation for this energy imbalance is the presence of an azimuthal magnetic field larger than $\sim 4.7\,\mathrm{T}$ that deflect the ions vertically. The uncertainties quoted represent 68% credible intervals.

## Introduction

Gas-puff is a member of the Z-pinch configuration, in which a column of supersonic gas is injected into the volume between the cathode and anode of a pulsed power generator. When the generator’s current ionizes and flows through the gas, an azimuthal magnetic field is generated that compresses the column radially until it reaches stagnation (the moment of maximum compression). Gas-puffs have been studied as potential sources of X-ray and neutrons^1–3^, and also for magneto-inertial fusion (MIF) studies^4^.

It is commonly believed that at stagnation the imploding plasma kinetic energy is rapidly thermalized (at the ion-ion energy equilibration time), and a big fraction of the kinetic energy is converted to thermal energy. Then, if the ion temperature is high enough, fusion reactions are produced (when deuterium is used as a working gas) and the thermal energy is converted to kinetic energy of the fusion product. In the case of radiation sources, the ions transfer their thermal energy to the electrons (at the ion–electron energy equilibration time), which in turns lose their energy through ionization and radiation^1,5,6^. However, in this work we show that this classic picture is not always the case and the physics at stagnation is more complex.

Furthermore, there are other processes at stagnation that are not fully understood. For example, acceleration of ions to energies larger than the driver voltage are usually observed; many theories have been suggested, but the acceleration mechanism still remains a source of controversy^7^. Also, the true ion temperature have been found difficult to measure. Maron et al.^8^ showed that the value obtained with Doppler spectroscopy can be several times higher than the real value, and that it represents all the hydrodynamic motion in the plasma rather than its thermal motion. This shows that more diagnostics and accurate data analysis are needed to fully understand the physics at stagnation.

The Thomson scattering (TS) technique has proven to be a powerful tool for diagnosing high energy density plasmas. With this technique, it is possible to estimate the electronic temperature (${\mathrm{T}}_{\mathrm{e}}$), ionic temperature (${\mathrm{T}}_{\mathrm{i}}$), electronic density (${\mathrm{N}}_{\mathrm{e}}$), plasma velocity (${\mathrm{V}}_{\mathrm{p}}$), ionization state ($\mathrm{Z}$) simultaneously^9–11^. This technique collects light scattered by the electron density fluctuation at a certain volume when a probing laser interacts with the plasma. The collected spectrum shape carries information about the plasma parameters. Its main advantage over other spectroscopy techniques is that it can measure locally from a well determined volume and is independent of broadening mechanisms such as the Stark or Zeeman effect. However, the large number of parameters $({\mathrm{T}}_{\mathrm{e}}, {\mathrm{T}}_{\mathrm{i}}, {\mathrm{N}}_{\mathrm{e}}, {\mathrm{V}}_{\mathrm{p}},\mathrm{ Z})$ and the complicated dependency of the mathematical model with the parameters (see Eq. 2) make it difficult to estimate simultaneously all parameters with their associated uncertainty. The conventional method of analysis involves several fixed parameters which are obtained from complementary diagnostics or by assumptions from previous experiments. The best fit is found by minimizing the chi-squared, while the uncertainty is estimated using a Monte Carlo-like method^9,12^. However, this point-estimate approach does not guarantee to reach the global minimum, especially in cases when the fit is multimodal or when different combinations of parameters can fit well the experimental data (resulting in a similar chi-squared), which is called in the literature as ‘unstable function’^13^. To deal with these cases is important to have a more general view of the parameter distribution allows us to determine how much each parameter influences the spectrum's shape and the correlation between parameters^14^.

The use of Bayesian inference provides us with the formalism to find the joint probability distribution of the model parameters inferred from the experimental data in a rigorous and quantitative way. The Bayesian approach has become a popular and versatile tool used for data analysis in various fields of physics^15^ and, more recently, in high-energy–density experiments^16–18^. The main advantage of the Bayesian inference over the fitting methods is that through the joint probability distribution of the model parameters, it is possible to know the uncertainty of each parameter and provide a greater vision of the behavior of the model and correlations between them. In addition, it allows evaluating which parameter needs to be accurately measured by supplementary diagnostics to restrict the model and thus make the implemented technique robust^13,19^.

In this paper, we implement the Thomson scattering technique in combination with Bayesian inference to estimate the most likely parameters given the experimental data, its probability distribution, the associated uncertainty, and the correlation between parameters. We find that at the beginning of stagnation, plasma is weakly collisional and plasma interpenetration occurs not thermalizing as quickly as it is commonly believed. Furthermore, we find that the ion energy at the periphery is much larger than the ion energy at the axis, even if we consider the radiation losses and the energy transfer to the electrons, there is still energy unaccounted for.

## Results and discussion

**Figure 1:**
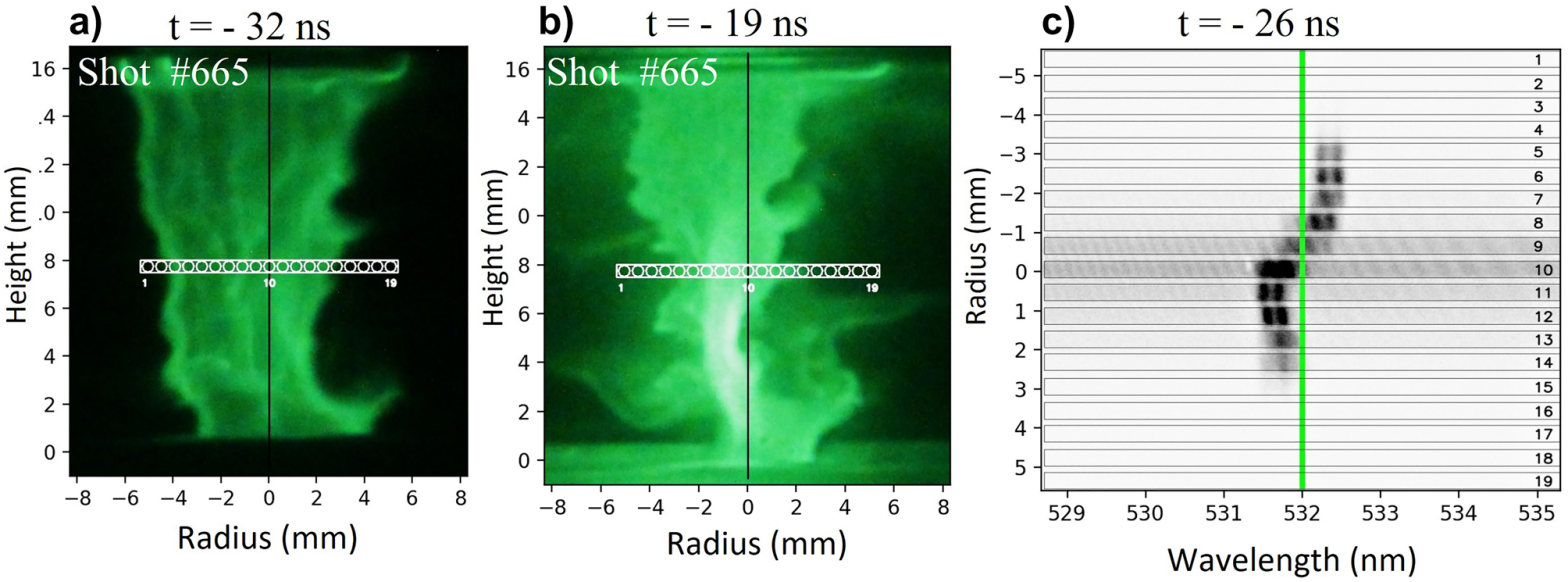
Raw data for shot 665. (**a**,**b**) show XUV pinhole images near to stagnation time (− 32 ns and − 19 ns, respectively). The white circles represent the collection volume for each of the 19 fibers used to collect light, and the black vertical line represents the chamber center. **c** The scattering spectra recorded at − 26 ns, the green vertical line shows the position of laser probe. Notice that negative and positive radii here represent position at the left and right of the axis, respectively.

Figure 1 shows raw data for shot 665. Thomson scattering data taken 26 ns before stagnation (-26 ns) is shown in Fig. 1c, while in Fig. 1a,b we show XUV pinhole images taken right before (-32 ns) and after (-19 ns) the Thomson data for ion acoustic waves (IAW) feature. The white circles represent the collection volume for each of the 19 fibers used to collect light along the radius. We see well-defined edges in Fig. 1a representing the boundary between plasma and vacuum. In addition, small perturbations due to the magneto-Rayleigh–Taylor (MRT) instabilities are seen, which evolve rapidly in time forming large spikes as observed in Fig. 1b. It is important to note that one of these spikes is located between fibers number 12 and 14. Data analysis from these fibers can be very difficult because MRT instabilities carry matter radially and axially at high velocity, and also because of the large density gradients which broaden the TS spectrum^9^. In Fig. 1c each of the 19 fibers are plotted along the vertical axis and represents the radial direction in the plasma, while the horizontal axis is the spectral direction. A first qualitative inspection of the raw data reveals information from the plasma which helps us to support the analysis. In the first place, it is observed that the pinch symmetry axis is centered on fiber 9 since the spectrum on this fiber has the smallest shift from the laser probe (532 nm). This coincides with XUV imaging data observed in Fig. 1a,b. We detect light from 10 fibers, which tell us the plasma radius by this time is $\mathrm{r}\approx 2.5\,\mathrm{mm}$. Red-shift Doppler is observed between fibers 5 to 8while blue shift is observed between fibers 10 to 14. The opposite Doppler shift is explained as the plasma velocity along the scattering vector ( $\overrightarrow{\mathrm{k}})$ points in the opposite directions on each side of the plasma column. In our case, since the scattering angle $\theta =90^\circ$ and under the assumption that the velocity is mainly radial, then ${\mathrm{v}}_{\mathrm{r}}= {\mathrm{v}}_{\mathrm{k}}/\mathrm{cos}(45^\circ )$.

The double peak observed in most of the fibers is characteristic of the ion feature in TS. However, it presents variations in its structure from fiber to fiber, indicative of changes in the plasma parameters. For example: in fiber 9 the spectrum broadens, and it is not possible to distinguish the characteristic IAW peaks. In fibers 13 and 14, it is observed that the right peak of the IAW is taller than the left one, which accounts for the presence of electron drift velocities and should be considered in the model. In addition, the Doppler shift for these fibers is less than observed in fiber 12. These last two effects account for the change in plasma dynamics due to MRT instability.

Before performing a quantitative analysis of the spectrum from each fiber, we estimated the velocity and electron density using XUV imaging and interferometry, respectively. This helps to choose the range of the prior distribution for the corresponding parameters , as it will be described later on. We find that during the last stage of the implosion the velocity increases approximately from $50\mathrm{ km}/\mathrm{s}$ to $380\mathrm{ km}/\mathrm{s}$ in $\Delta t \approx 50 ns$. On the other hand, from Mach Zehnder interferometry the density expected at -26 ns is between $1\mathrm{x}{10}^{19}{\mathrm{cm}}^{-3}$ and $1\mathrm{x}{10}^{20}{\mathrm{cm}}^{-3}$ (see supplementary material).

**Figure 2:**
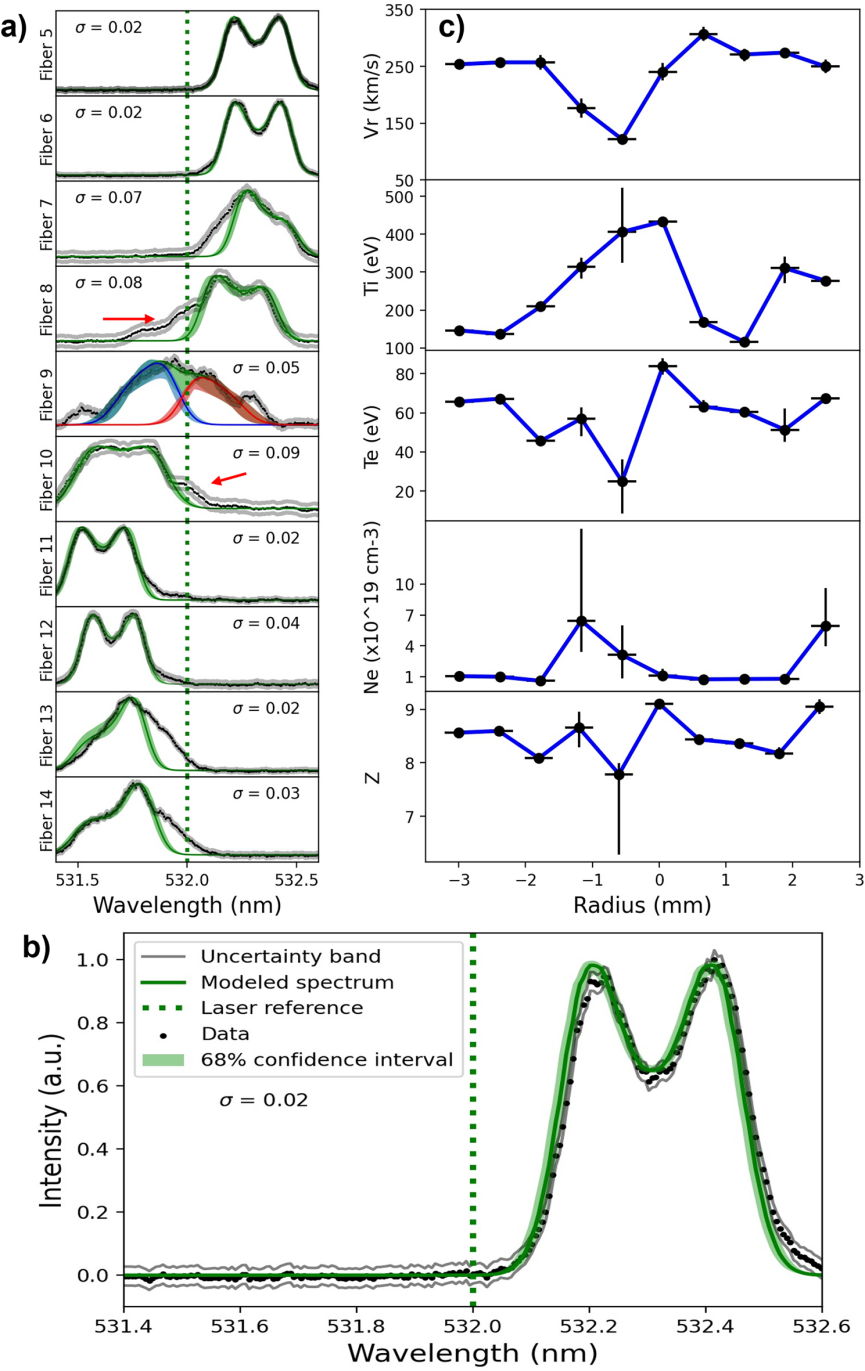
(**a**) Normalized spectral profiles with respect to the maximum value for each fiber (black points), uncertainty band (gray lines) and the modeled spectrum (green line) which represent the median values for all spectra with their corresponding 68% credible interval (green band). (**b**) We show the spectrum from fiber 5 to better observe the accuracy of the spectrum modeled with respect to data. (**c**) The median of inferred plasma parameters with a credible interval of 68% at different radii.

The quantitative results obtained from TS are summarized in Fig. 2. In Fig. 2a we show the experimental profiles (black points) extracted from the raw data normalized with the maximum value of each fiber. While laser reference is shows as a green dotted line. The spectrum modeled using the median values for all spectra with their corresponding 68% credible interval is given by a green band and the uncertainty bands determined by Bayesian inferences is represented by gray lines. It can be seen that the spectrum modeledfor fibers 5, 6, 11, and 12 show excellent agreement with the experimental data (are indistinguishable within the uncertainty). On the other hand, fibers located around the center (7, 8, and 10) present a tail (indicated in Fig. 2a by the red arrow). These tails are closer to the laser wavelength and even present a Doppler shift in the opposite direction with respect to the bulk plasma, indicative of a plasma moving in the opposite direction. As we show below, the plasma is weakly collisional and this signal comes from counterpropagating plasma that reaches the center moments before the bulk plasma, then they interact and interpenetrate, thus being detected on adjacent fibers.

Given the observation mentioned above, fiber 9 (center) was modeled as two identical counterstreaming plasmas (red and blue lines) with an electron drift velocity of 500 km/s. The sum of these counterstreaming signals is closely to the experimental data confirming the interpenetration of flows from opposite directions.

Finally, for fibers 13 and 14 we observe the following: first, the TS signal presents a tail closer to the laser wavelength indicating a plasma moving at a slower speed; second, the IAW peaks are not symmetric indicating an electron drift with respect to the ions. Considering the last point, a drift velocity of ~ 500 km/s was necessary for most of the modeled spectrum to be within the band of the given uncertainty. In this case (fiber 13 and 14), the changes in the spectrum's shape are attributed to the MRT instability. However, further analysis of the MRT instability using Thomson scattering is outside the scope of this paper and will poste be addressed in future work. Therefore, in the following discussion, we will only focus on analyzing the most central fibers where no instabilities are observed.

**Figure 3:**
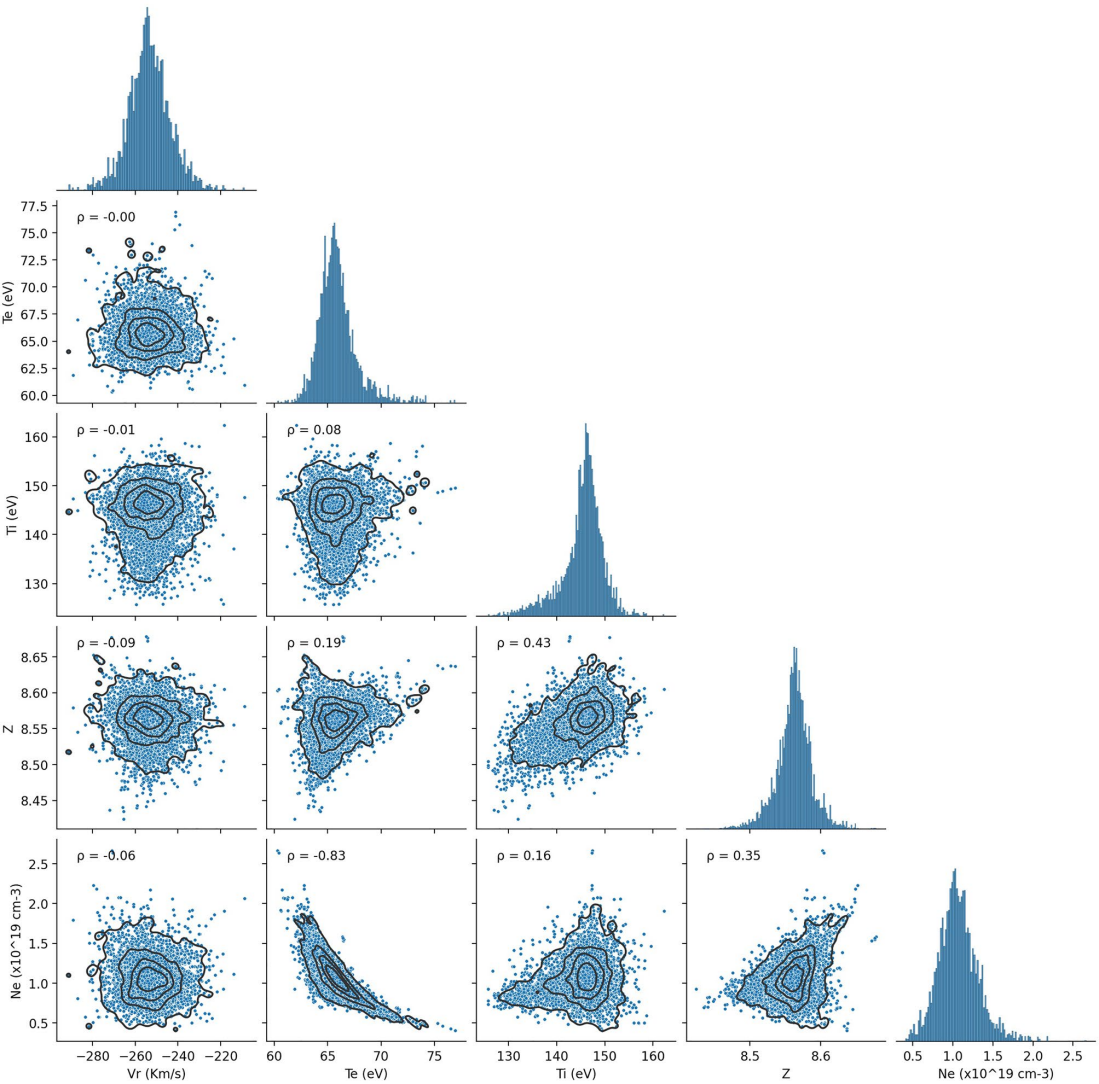
Corner plot the 5 infered parameters from fiber 5. The on-diagonal plots show the marginal posterior distribution and the off-diagonal plots are the joint posterior distributions for pair-wise combinations of parameters. Here, $\rho$ is the Pearson correlation coefficient.

An example of the Bayesian analysis output is shown in Fig. 3 for fiber number 5. The marginal posterior distributions for each parameter are shown and the off-diagonal plots are the joint posterior distributions for pair-wise combinations of parameters. We observe that the sample points appear to distributed approximately as a multi-variate normal distribution, It can also be seen that almost pair-wise combinations of parameters have null or low correlation (Pearson's coefficient < 0.4). However, on the Ne vs Te plot the points are grouped with a tendency to decrease the electron density as the electronic temperature increases. It shows that both variables are strongly anti-correlated with a Pearson coefficient of − 0.83, and implies that combinations with a specific trend between these parameters (Ne, Te) describe the data for the given model. Thus, since these parameters are anti-correlated, it is recommended for future work to implement complementary and simultaneous diagnostics to measure Ne or Te that allow a better choice of the prior distribution , for example by simultaneously measuring the electron feature in TS.

Applying the procedure mentioned above for each fiber, the radial variation of the inferred parameters can be obtained as shown in Fig. 2c. Each point represents the median of the posterior distribution, and the vertical error bars are the 84.2% and 15.8% percentiles, which represent the 68% credible intervals. The horizontal error bar is taken as the spatial resolution of each fiber (fiber size times the optical magnification).

**Figure 4:**
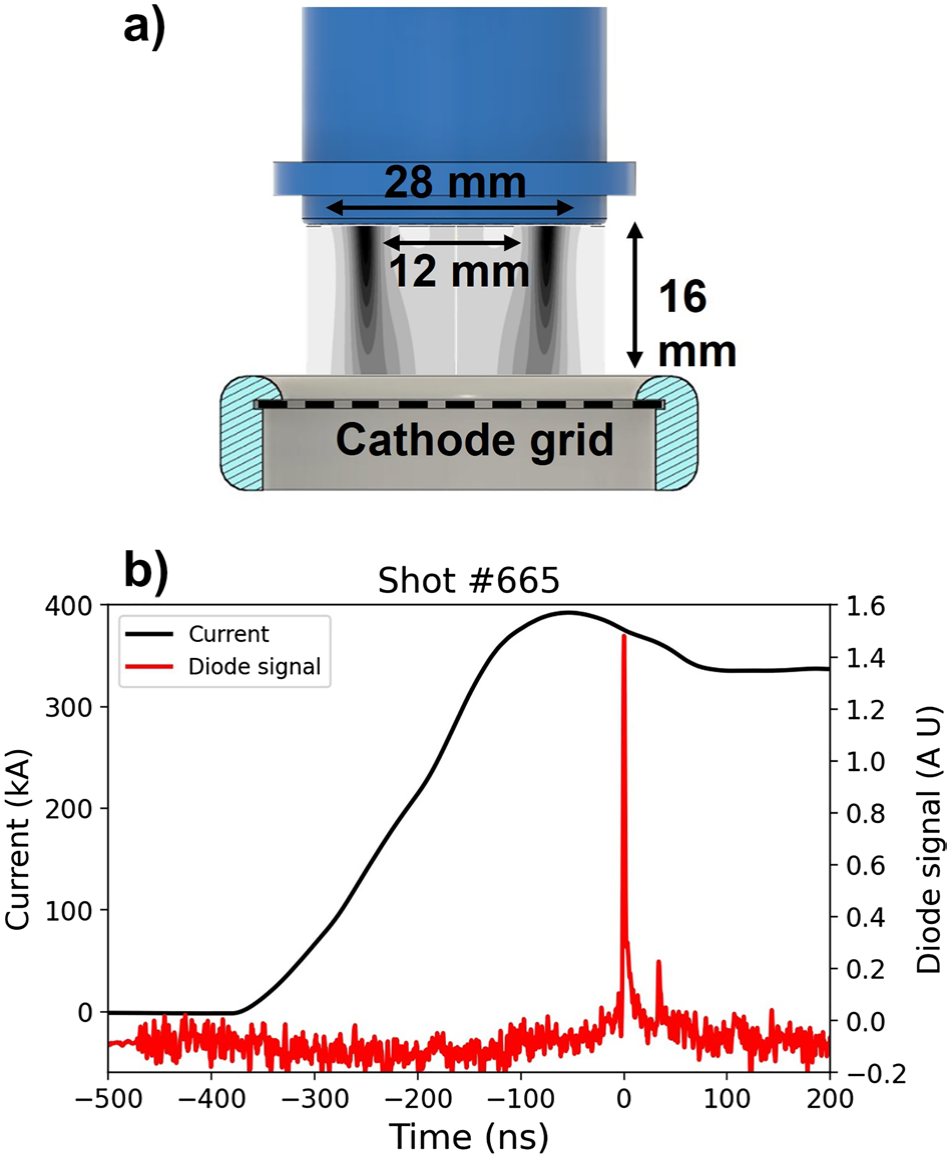
(**a**) Schematic of the gas-puff configuration. Injector is shown in blue and is located at the anode, gas is injected towards the cathode which is composed of a wire mesh. The gas is shown in grey scale—obtained from a computational fluid dynamics simulation—between the anode and cathode. (**b**) the experimental current trace is shown in black and the signal of a Be filtered diode in red for the shot number 665. Here zero time indicate stagnation given by maximum emission of the diode’s signal.

As shown in Fig. 2c, at the periphery the plasma velocity and electron temperature are ${253.7}_{-8.8}^{+9.5}\,\mathrm{ km}/\mathrm{s}$ and ${65.7}_{-1.3}^{+1.7}\,\mathrm{ eV}$ , respectively, which decreases to ${127.8}_{-7.8}^{+7.9}\,\mathrm{ km}/\mathrm{s}$ and ${24.9}_{-16.2}^{+11.4}\,\mathrm{ eV}$ at the center. An inverse behavior is observed for the ion temperature and electron density, which increase from ${146.1}_{-4.3}^{+2.8}\,\mathrm{ eV}$ and $\left({1.1}_{-0.2}^{+0.3} \right)\times {10}^{19}{\mathrm{cm}}^{-3}$, respectively, at the periphery to ${406.1}_{-81.0}^{+116.3}\,\mathrm{ eV}$ and $\left({3.1}_{-2.3}^{+2.9}\right)\times {10}^{19}{\mathrm{cm}}^{-3}$ at the center. While no large changes in the ionization state is observed, measuring ~ 8.5 across the radius. It can be seen that the velocity and electron density values agree with the values estimated from the MCP and interferometry data, which helps us to validate the results obtained (see supplementary material). The results reveal a significant difference in the total energy per ion ( ${\mathrm{E}}_{\mathrm{T}}= 1/2 \left({\mathrm{m}}_{\mathrm{i}}{\mathrm{V}}_{\mathrm{r}}^{2}+3{\mathrm{k}}_{\mathrm{B}}{\mathrm{T}}_{\mathrm{i}}\right)$) as a function of the radius. At the periphery, the kinetic component has a ${0.98}_{-0.09}^{+0.10}$ fraction of the total energy ${13.6}_{-0.9}^{+1.0}\,\mathrm{ keV}$. However, at the center, the thermal component increase and the kinetic energy decrease reaching a ${0.84}_{-0.14}^{+0.15}$ fraction of the total energy per ion (${3.7}_{-0.4}^{+0.4}\,\mathrm{ keV})$. The small increase in the thermal component from ${146.1}_{-4.3}^{+2.8}\,\mathrm{ eV}$ at the periphery (r = 3 mm) to ${406.1}_{-81.1}^{+116.3}\,\mathrm{ eV}$ at the center is consistent with the flared tails in the profiles in Fig. 2a previously discussed, where the plasma at the center is able to interpenetrate for some length while transferring some of its kinetic energy to thermal energy. However, the interpenetration does not explain the missing energy at the center. Some loss mechanisms to consider are the energy transferred to the electrons and the energy lost as radiation. Nonetheless, our results indicate that these mechanisms cannot account for the missing energy. On one hand, the energy transfer to the electrons is small considering the data presented in Fig. 2c. On the other hand, the X-ray emission detected by a Be-filtered diode does not measure strong emission at this time either (-26 ns prior to stagnation, as shown in Fig. 4b. Even if we consider the total X-ray yield above 1 keV detected by the Be-filtered diode it is much less than 1 eV per ion.

Therefore, we conjecture that the missing energy may be stored in an axial component of the velocity, which in our configuration cannot be detected because the k-vector lies on the radial plane. Notice that this behavior has been observed previously in tungsten wire arrays^20^, where the magnetic field is advected by the incoming plasma flows and it deflects the ions axially. Magnetic field advection has also been measured in gas-puffs using Zeeman spectroscopy, values of ~ 4 T were detected inside the plasma^21^. This evidence supports the conjecture presented above. Assuming this was the case here, the magnetic field necessary to deflect the ions can be estimated by $\mathrm{B}= {\mathrm{m}}_{\mathrm{i}}{\mathrm{V}}_{\mathrm{r}}\mathrm{f}/\mathrm{eZr}$. Where ${\mathrm{m}}_{\mathrm{i}}$ is the ionic mass, ${\mathrm{V}}_{\mathrm{r}}$ is the velocity at the periphery of the plasma, $\mathrm{e}$ is the electron charge, $\mathrm{Z}$ ionization state, $\mathrm{r}$ is the radius of the plasma, and $\mathrm{f}= \sqrt{{\mathrm{V}}_{\mathrm{r}}^{2}/\left({\mathrm{V}}_{\mathrm{r}}^{2}-{\mathrm{V}}_{\mathrm{r}=0}^{2}\right)}$ is a correction factor that takes into account the ion deflection according to the measured at the center ${\mathrm{V}}_{\mathrm{r}=0}$. Using $\mathrm{Z}=8.5\pm 0.1,{\mathrm{V}}_{\mathrm{r}}={253.7}_{-8.8}^{+9.5}\,\mathrm{ km}/\mathrm{s}, {\mathrm{V}}_{\mathrm{r}=0}={127.8}_{-7.8}^{+7.9}\,\mathrm{ km}/\mathrm{s and r}=2.5\pm 0.2\,\mathrm{mm}$, the azimuthal magnetic field estimated is ${4.7}_{-0.3}^{+0.4}\,\mathrm{ T}$. This field can easily be achieved in our configuration, where the maximum expected field according to Ampere’s law is 32 T calculated at the same radius and maximum current 400 kA. So, it seems possible that a ${4.7}_{-0.3}^{+0.4}\,\mathrm{ T}$ field be present near the axis that deflects the ions vertically.

To evaluate the interpenetration, we computed the ion-ion mean free path (${\mathrm{l}}_{\mathrm{i}-\mathrm{i}}={\mathrm{V}}_{\mathrm{i}-\mathrm{i}}/{\upnu }_{\mathrm{i}-\mathrm{i}})$, where ${\mathrm{V}}_{\mathrm{i}-\mathrm{i}}$ is the relative ion velocity, and ${\upnu }_{\mathrm{i}-\mathrm{i}}$ is the ionic collision frequency estimated according to Rambo et al.^22^. Also, to evaluate the advection of a magnetic field suggested above, the magnetic Reynolds number was estimated as ${\mathrm{R}}_{\mathrm{em}}={\mathrm{rV}}_{\mathrm{r}}/\upeta$ , where ${\mathrm{V}}_{\mathrm{r}}$ is the radial velocity of the plasma, r is the length of the flux path, and $\eta$ is the transverse Spitzer resistivity^5^. The results are summarized in Table 1 for three different radial position.

**Table 1:** Plasma parameters for different radius.

$\mathrm{r}$±0.2 (mm)	${\mathrm{V}}_{\mathrm{r}}$ (km/s)	${\mathrm{N}}_{\mathrm{e}}$ ${\mathrm{x}10}^{19}$ (cm^−3^)	$\mathrm{Z}$	${\mathrm{T}}_{\mathrm{e}}$ (ev)	${\mathrm{T}}_{\mathrm{i}}$ (ev)	${\uplambda }_{\mathrm{i}-\mathrm{i}}$	${\uplambda }_{\mathrm{e}-\mathrm{i}}$	${\upnu }_{\mathrm{i}-\mathrm{i}}$ ${\mathrm{x}10}^{7}$ (1/s)	${\mathrm{l}}_{\mathrm{i}-\mathrm{i}}$ (μm)	$\upeta$ m^2^/s	${\mathrm{R}}_{\mathrm{em}}$
-1.8	${257.1}_{-8.8}^{+9.5}$	${0.6}_{-0.1}^{+0.1}$	${8.1}_{-0.1}^{+0.1}$	${45.6}_{-2.2}^{+2.4}$	${209.1}_{-1.7}^{+0.7}$	${9.4}_{-0.1}^{+0.1}$	${5.0}_{-0.1}^{+0.1}$	${6.5}_{-1.3}^{+1.3}$	${7900}_{-1610}^{+1610}$	${10.8}_{-0.1}^{+0.1}$	${42.9}_{-4.8}^{+4.8}$
− 1.2	${176.7}_{-16.6}^{+16.5}$	${6.4}_{-3.0}^{+8.9}$	${8.7}_{-0.4}^{+0.3}$	${57.1}_{-9.1}^{+5.6}$	${314.0}_{-31.2}^{+23.1}$	${7.4}_{-0.3}^{+0.7}$	${4.1}_{-0.3}^{+0.7}$	${202}_{-113}^{+287}$	${175}_{-99}^{+249}$	${6.7}_{-0.2}^{+0.2}$	${31.6}_{-5.3}^{+5.3}$
− 0.6	${127.8}_{-7.8}^{+7.9}$	${3.1}_{-2.2}^{+2.8}$	${7.8}_{-1.5}^{+0.2}$	${24.9}_{-16.2}^{+11.4}$	${406.1}_{-81..1}^{+116.3}$	${6.9}_{-0.8}^{+0.9}$	${3.3}_{-1.1}^{+0.9}$	${163}_{-153}^{+152}$	${156}_{-148}^{+147}$	${16.9}_{-2.0}^{+1.4}$	${4.5}_{-1.6}^{+1.6}$

Where ${\uplambda }_{\mathrm{i}-\mathrm{i}}$ and ${\uplambda }_{\mathrm{e}-\mathrm{i}}$ are the ion-ion and electron–ion coulomb logarithm^23^. Here, we use two plasma species with the same parameters given at that position.

These results confirmed that plasma interpenetration is possible. Furthermore, a significant spatial dependence of the ion mean free path is observed as it approaches the center. At the periphery, the plasma appears to be collisionless $({\mathrm{l}}_{\mathrm{i}-\mathrm{i }}={7.9}_{-1.6}^{+1.6}\,{\text{mm}})$ . However, as it approaches the center, it rapidly changes to a quasi-collisional $({\mathrm{l}}_{\mathrm{i}-\mathrm{i }}={175}_{-99}^{+249}\,\upmu\text{m})$ and then to $({\mathrm{l}}_{\mathrm{i}-\mathrm{i }}={156}_{-148}^{+147}\,\upmu\text{m})$ at the center.

We also find that the magnetic Reynolds number is ${Re}_{m}>30$ at $r>1mm$, which also suggests that some magnetic field could be advected as the plasma column compresses deflecting the ions vertically.

## Summary and conclusions

Bayesian inference has been successfully implemented to diagnose a gas-puff plasma close to stagnation time using the collective Thomson scattering technique. The model was used to infer five plasma parameters (velocity, ion temperature, electron temperature, density, and ionization state). Plasma velocity and density were validated with MCP imaging and interferometry measurements. This procedure reveals an anti-correlation between temperature and electron density. In future works, the electronic feature of the Thomson scattering to be measured to determine electron density. In this way, it becomes possible to choose prior distribution better to obtain electronic temperature results with more precision.

The Analysis of the inferred parameters reveals important new information about the implosion dynamics for times close to stagnation. The measurements show a substantial change in the plasma collisionality within a few millimeters, changing from a weakly collisional regime at the periphery to a collisional regime on approaching the axis. In addition, data suggest the existence of an azimuthal magnetic field inside the pinch formed by the advection of the induced field, which deflects the ions in the axial direction. To allow this effect, a field of 4.7 T would be sufficient to deflect the ions.

More experiments are underway to unravel the dynamics of interpenetration and deflection of ions in a gas-puff. In future experiments, multi-angle Thomson scattering and Zeeman spectroscopy techniques will be used simultaneously to infer the radial and axial plasma velocity components as well as the distribution of the magnetic field. These studies will help delve deeper into the processes governing the implosion of the gas-puff.

## Methods

### Experimental setup

The experiment was carried out on the Llampudken pulsed power generator^24^ located at the Instituto de Física of the Pontificia Universidad Católica de Chile. Llampudken is capable of delivering a peak current of ~ 400kA at ~ 200 ns rise time (10% to 90%) when charged to 22 kV per capacitor. A characteristic current trace is shown in Fig. 4b. We used an argon load injected by a ~ 4.5 Mach annular gas-puff injector whose outlet has a 12 mm internal and 28 mm external diameter, see Fig. 4a. The injector is placed on the anode, which is located 16 mm away from a mesh cathode, and it produces a gas load of ~ 4 µg/cm line density in order to reach maximum compression right after peak current.

**Figure 5:**
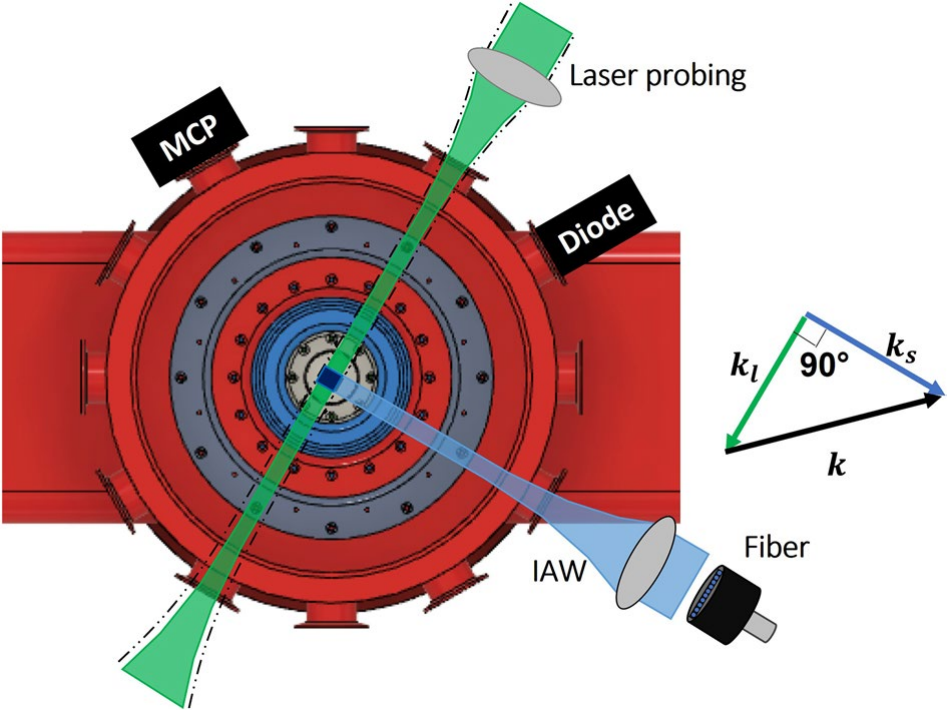
Thomson-scattering and complementary diagnostic configuration on Llampudken. The laser was focused by a 1500 mm focal length lens to a $50\mu m$ diameter beam waist spot. Light scatterd by the plasma was collected at 90° using a 100 mm focal length lens focused onto a fiber array. On the right we show the direction of the ${\varvec{k}}$ vector given by the difference between the scattering ${{\varvec{k}}}_{{\varvec{s}}}$ and laser ${{\varvec{k}}}_{{\varvec{l}}}$ wavevectors.

A Thomson scattering diagnostic was implemented using a 532 nm Nd-YAG laser (EKSPLA NL310) which can produce a pulse up to 1 J with 4 ns full width half maximum (FWHM). The polarization was oriented vertically by a half-wave plate to maximize the scattered light in the collection direction (see Fig. 5). The laser beam was focused with a 1500 mm focal length lens to a ~ 50 µm diameter beam waist. Brewster windows were used at the chamber input and output to reduce energy losses and avoid reflections inside the chamber. Light was collected 90° with respect to the laser beam using a 100 mm focal length lens focused onto a multimode linear fiber array with a magnification of ~ 2.1. The fiber array has nineteen 200 µm diameter fibers align along the laser beam direction. The Thomson scattering collection volume is given by the cross-sectional area of the laser times the length of the optic collection volume ($\mathrm{l}=2.1\times 200\,\mathrm{\mu m}=420\,\mathrm{\mu m}$). The other end of the fiber is coupled to a 500 mm focal length spectrometer (SpectraPro HRS-500) with a 50 µm entrance slit and 2400 l/mm grating. The optical system is aligned to spatially resolve each fiber, and achieve a spectral resolution of 0.1 nm at 532 nm. The spectra were recorded with a gated ICCD (Stanford 4picos) using an 8 ns window to completely pick up the laser pulse.

Complementary diagnostics were used to study the plasma evolution. To provide information about the compression and uniformity of the pinch, extreme ultraviolet (XUV) self-emission imaging is performed using unfiltered 100 µm pinhole and a four-frame micro-channel plate camera (MCP) with ~ 4 ns temporal resolution. Mach–Zehnder interferometry is performed using a second laser (1 mJ at 532 nm and 4 ns FWHM) to estimate the electron density at early times. The interferograms were recorded digitally on a canon camera (EOS Rebel T3i). We also used a 25 µm Be filtered diode (AXUV HS5) to measure the stagnation time and X-ray yield (> 1kev).

### Thomson scattering

Thomson Scattering consists of elastic scattering of radiation (e.g. a laser) by free charged particles present in the plasma. However, since the electrons are much lighter than the ions in the plasma, the measured Thomson spectrum is mainly produced by the electrons as their acceleration in the laser field is much larger compared to the ions^25^. Nevertheless, the electrons' response is determined not only by the laser's electric field but also by the presence of more electrons and ions that shield each other. Specifically, we need to compare the wavelength associated with the scattering wavevector ($\uplambda =2\uppi /\mathrm{k}$ where $\mathrm{k}\approx 2{\mathrm{k}}_{\mathrm{l}}\mathrm{sin}(\uptheta /2))$ with the shielding length (Debye length, ${\uplambda }_{\mathrm{De}}= \sqrt{{\mathrm{N}}_{\mathrm{e}}{\mathrm{e}}^{2}/{\upvarepsilon }_{0}{\mathrm{k}}_{\mathrm{B}}{\mathrm{T}}_{\mathrm{e}}}$), with θ is the angle between the laser path and the collection optics. If $\uplambda <{\uplambda }_{\mathrm{D}}$ , the electrons are noncorrelated, so they are randomly distributed due to thermal fluctuations, and the scattered light is incoherent. However, if $\uplambda >{\uplambda }_{\mathrm{D}}$, collective behavior is observed so that the scattered light can be coherent due to the presence of local fields produced by plasma waves. The parameter alpha ($\mathrm{\alpha }= 1/\mathrm{k}{\uplambda }_{\mathrm{De}})$ determines the relationship between both lengths. If $\mathrm{\alpha }<1$ the regime is non-collective and if $\mathrm{\alpha }>1$ the regime is collective. A comprehensive review of both collective and non-collective Thomson scattering can be found in Ref.^25^. We find that for the plasma parameters in our experiment (see Fig. 2b), scattering is in the collective regime.

In the non-relativistic and collisionless limit, the total scattered power (${\mathrm{P}}_{\mathrm{s}}$) per unit solid angle ($\mathrm{d\Omega }$) and scattered frequency ($\mathrm{d}{\upomega }_{\mathrm{s}}$):

1 $${\text{P}}_{\text{s}} {{\text{d}}}{\Omega}{\text{d}}{\omega }_{\text{s}}= {{\text{P}}_{\text{l}}}{{\text{r}}_{0}^{2}}{{\text{Ld}}\Omega}\frac{{{\text{d}}{\omega}}_{\text{s}}}{2\pi } \left(1+\frac{2\omega }{{\omega }_{\text{l}}}\right)[{\widehat{\text{k}}}_{\text{s}} {\times} {(\widehat{\text{k}}}_{\text{s}} {\times} {\widehat{\text{E}}}_{\text{l}})]{\text{N}}_{\text{e}}\text{S}\left(\textbf{k},\omega \right)$$

where ${P}_{l}$ is the average incident power, ${\mathrm{r}}_{0}$ is the classical electron radius, $\mathrm{L}$ is the length of scattering volume, ${\mathrm{N}}_{\mathrm{e}}$ is the electron density, ${\widehat{\mathrm{E}}}_{\mathrm{l}}$ is the electric field direction, $\upomega = {\upomega }_{\mathrm{s}}-{\upomega }_{\mathrm{l}}$ (${\upomega }_{\mathrm{s}}$ and ${\upomega }_{\mathrm{l}}$ are the frequencies of scattered light and laser probe), $\mathbf{k}={\mathbf{k}}_{\mathbf{s}}-{\mathbf{k}}_{\mathbf{l}}$ (${k}_{s}$ and ${\mathrm{k}}_{\mathrm{l}}$ are the wavevectors of scattered light and laser probe) and $\mathrm{S}\left(\mathbf{k},\upomega \right)$ is the scattering form factor given by:

2 $$\mathrm{S}\left(\mathbf{k},\upomega \right) = \frac{2\uppi }{\mathrm{k}}{\left|1+\frac{{\upchi }_{\mathrm{e}}}{\upvarepsilon }\right|}^{2}{\mathrm{f}}_{\mathrm{e}}\left(\frac{\upomega }{\mathrm{k}}\right) + \frac{2\uppi }{\mathrm{k}}{\sum_{\mathrm{j}}\frac{{\mathrm{Z}}_{\mathrm{j}}^{2}{\mathrm{N}}_{\mathrm{j}}}{{\mathrm{N}}_{\mathrm{e}}}\left|\frac{{\upchi }_{\mathrm{e}}}{\upvarepsilon }\right|}^{2}{\mathrm{f}}_{\mathrm{i}}\left(\frac{\upomega }{\mathrm{k}}\right)$$

here $\upvarepsilon =1+{\upchi }_{\mathrm{e}}+\sum_{\mathrm{j}}{\upchi }_{\mathrm{ij}}$ is the dielectric function, ${\upchi }_{\mathrm{e}}$ and ${\upchi }_{\mathrm{i}}$ are the electron and ion susceptibility, ${\mathrm{Z}}_{\mathrm{j}}$ and ${\mathrm{N}}_{\mathrm{j}}$ are the ionization state and density of j-th ion population (subscript j), ${\mathrm{f}}_{\mathrm{e}}$ and ${\mathrm{f}}_{\mathrm{i}}$ are the normalized one-dimensional Maxwellian electron and ion distribution functions projected along $\mathbf{k}$, respectively. The two terms in Eq. (2) take into account the shielding around the electrons (term at the left) and the shielding around the ions (term at the right). These terms give rise to the so-called electron and ion features, which correspond to scattering from longitudinal electron plasma waves (EPW) and ion acoustic waves. Typically, the ion feature is two orders of magnitude narrower than the electron feature in the wavelength domain, so in principle, they are well distinguishable by choosing a spectrometer with high enough spectral resolution^26^.

### Bayesian analysis

In the Bayesian approach to data analysis, the viability of a hypothesis "H" is assessed by calculating the probability of H given the observed data "X" and prior knowledge of assumptions or physical constraints "I". H can be a combination of a parameter (${\uptheta }_{1},{\uptheta }_{2},\dots .{\uptheta }_{\mathrm{N}})$ and possible mathematical models (${\mathrm{M}}_{1},{\mathrm{M}}_{2},\dots .{\mathrm{M}}_{\mathrm{N}})$ or a series of parameters for a given model, which depends on the problem to be analyzed^15^. In our case, the model is described by Eq. (2) which parameterized by ${\varvec{\uptheta}}=({\mathrm{T}}_{\mathrm{e}}, {\mathrm{T}}_{\mathrm{i}}, {\mathrm{N}}_{\mathrm{e}}, {\mathrm{V}}_{\mathrm{p}},\mathrm{Z}$), that is, $\mathrm{S}\left(\mathbf{k},\upomega ;{\varvec{\uptheta}}\right)$. So, we want to find the probability of this set of parameters ${\varvec{\uptheta}}$, given the experimental data **X** and our prior knowledge I. Using Bayes's theorem, this probability can be written as:

3 $$\mathrm{P}\left(\uptheta |\mathrm{X},\mathrm{I}\right)= \frac{\mathrm{P}(\mathrm{X}|\uptheta ,\mathrm{I})\mathrm{P}(\uptheta |\mathrm{I})}{\mathrm{P}(\mathrm{X}|\mathrm{I})}$$

where $\mathrm{P}\left(\uptheta |\mathrm{X},\mathrm{I}\right)$ is known as the posterior distribution for the parameter set, $\mathrm{P}(\mathrm{X}|\uptheta ,\mathrm{I})$ represents the probability of observing the data **X**, given a set of model parameters, ${\varvec{\uptheta}}$, known as the likelihood.

The choice of the likelihood function is an important factor in Bayesian inference; this function contributes to the shape of the posterior distribution and therefore affects the uncertainty of the process^19^. The correct way to choose the likelihood function remains open since, in most cases, the implemented model is imperfect, and it is difficult to know the correlation in the measured signal^13^. A poor model and a poor choice of the likelihood function can introduce a bias in the inferred parameters combined with a low variance. Several authors have considered different likelihood functions in the context of high-energy–density physics^13,17,19,27^). In our case, for a first approach to the Bayesian analysis applied to Thomson cattering data, we consider a normal distribution described by Eq. (4), which we believe to be the most appropriate given that the model used (see Eq. 2) is robust and it hass been tested extensively^25^. However, the effect of different likelihood functions on the inference of plasma parameters given the mode and experimental data, could be studied in a future work.

4 $$\mathrm{P}\left(\mathrm{X}|\uptheta ,\mathrm{I}\right)= \prod_{\mathrm{i}=1}^{\mathrm{n}}\frac{1}{\sqrt{2\uppi }\upsigma }{\mathrm{e}}^{-\frac{1}{2{\upsigma }^{2}}{({\mathrm{S}}_{\mathrm{i}}(\uptheta )-{\mathrm{X}}_{\mathrm{i}})}^{2}}$$

Since in our case we do not know the correlation of the measured spectrum nor the experimental noise, we also find $\upsigma$ value as an additional parameter in the inference, i.e. six parameters $\left({\mathrm{T}}_{\mathrm{e}}, {\mathrm{T}}_{\mathrm{i}}, {\mathrm{N}}_{\mathrm{e}}, {\mathrm{V}}_{\mathrm{p}},\mathrm{Z },\upsigma \right)$^28^. The median of the $\upsigma$ infered in each case is shown in Fig. 2a. for each fiber.

However, it is important to mention that the assumption of a constant value for σ is strictly valid only when the noise is uncorrelated across the spectrum. In cases where the noise is correlated, using a constant sigma may lead to an underestimation of the credible interval. We are currently conducting a more rigorous analysis of the noise in the measurements to enhance the accuracy of our analysis. The results of this investigation will be presented in a future work.

The likelihood function is multiplied by the prior distribution $\mathrm{P}(\uptheta |\mathrm{I})$, which is important as it provides a way to bias the parameters based on prior knowledge. In our case, we use information from previous experiments operated under similar conditions using complementary diagnostics such as extreme ultraviolet (XUV) imaging and Mach–Zehnder interferometry. However, since the inherent jitter of the system can modify the plasma parameters in different shots and since we do not have enough shots to have a distribution for each parameter, we prefer to use a conserved approach where all values in a certain range have the same probability. That is, we choose a uniform distribution for each parameter listed in Table 2. Finally, $\mathrm{P}(\mathrm{X}|\mathrm{I})$ is known as marginal likelihood or evidence and is interpreted as a normalization constant that is not crucial for parameter estimation^15^. The marginal likelihood is important when selecting between models. It could have been used here, but it would require a more sophisticated sampling algorithm which is outside the scope of this paper.

**Table 2:** Prior distribution used in the inference.

Parameter	Prior distribution	Range
${\mathrm{V}}_{\mathrm{p}}$ (km/s)	Uniform	− 400 to 400
${\mathrm{N}}_{\mathrm{e}}$ (cm^−3^)	Uniform	${1\times 10}^{17}$ to ${9\times 10}^{19}$
${\mathrm{T}}_{\mathrm{e}}$(ev)	Uniform	10–150
${\mathrm{T}}_{\mathrm{i}}$(ev)	Uniform	50–1000
$\upsigma$	Uniform	0.001–0.5

Before implementing the Bayes code, the model for the scattering form factor $\mathrm{S}\left(\mathbf{k},\upomega ;{\varvec{\uptheta}}\right)$ was convolved with the optical system's instrumental response, and we created a look-up table for the ionization state (Z) using the radiative collisional code PrismSPECT^29^. This way, given ${\mathrm{T}}_{\mathrm{e}}$ and ${\mathrm{N}}_{\mathrm{e}}$, Z is found by interpolating from the nearest values in the table. The Bayes algorithm to calculate the posterior distribution was written on Python using the LMFIT and EMCEE libraries^30,31^ and using the Markov Chain Monte Carlo (MCMC) method with starting point the set of parameters obtained from a nonlinear least squares optimization.

### Supplementary Information

Supplementary Information.

## Data Availability

The data that supports the findings of this work are available from the corresponding author upon email request.

## Code availability

The python codes used in this work are available from the corresponding author upon email request.
