# Supplementary material for: Bayesian inference of plasma parameters from collective Thomson scattering technique on a gas-puff near stagnation
Source: Sci Rep. 2023 Aug 10;13:13002. doi: 10.1038/s41598-023-40014-x (PMC10415259; doi:10.1038/s41598-023-40014-x)
Supplement: Supplementary file 1 — Supplementary Information. [file 41598_2023_40014_MOESM1_ESM.docx]

**Supplementary material**

**Bayesian inference of plasma parameters from collective Thomson scattering technique on a gas-puff near stagnation**

*M. Escalona^1^, J. C. Valenzuela^1*^, G. Avaria^2^, F. Veloso^1^ and E. S. Wyndham^1^*

*^1^Instituto de Física, Pontificia Universidad Católica de Chile, Av. Vicuña Mackenna 4860*

*^2^Research Center on the Intersection in Plasma Physics, Matter and Complexity, P2mc, Comisión Chilena de Energía Nuclear, Casilla 188-D, Santiago, Chile*

**jcvalenzuela@fis.uc.cl*

**Alpha parameter**

In our experiment, when $\lambda_{l}=532 nm$ and $\theta=90^{\circ}$, the alpha parameter can be rewritten as $\alpha=8.05 x {10}^{-9} \sqrt{{N_{e}[{cm}^{-3}]}/{T_{e}[eV]}}$ ,which is graphically represented in Fig. 1 for typical values of electronic densities and temperatures found in gas-puffs. We find that for the plasma parameters in our experiment scattering is collective.


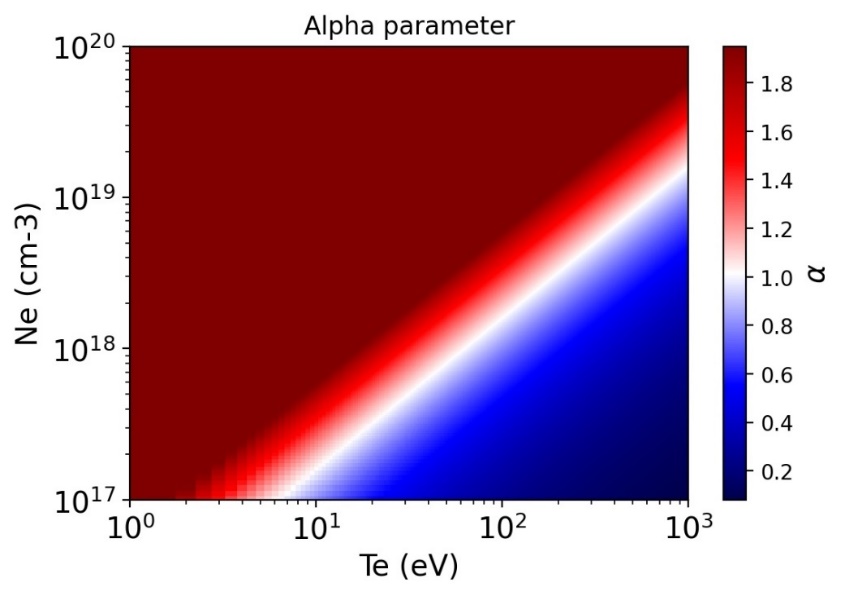


Supplementary figure 1. Colormap of the alpha parameter for typical values of electronic densities and temperatures of the experimental setup ($\lambda_{l}=532 nm$ and $\theta=90^{\circ}$). White color represents values with $\alpha=1$, while dark red and dark blue represent collective and non-collective regions, respectively.

**Complementary Diagnostics**

Before performing a quantitative analysis of the spectrum, we estimate the velocity and electron density using the XUV imaging and interferometry, respectively

This helps to constrain the prior parameters distribution and also to compare with the results obtained from TS. The XUV images were processed using a contour detection script on Python to find the outermost edge of the plasma. The results shown in Fig. 2(a) contains data from 19 shots, where each shot accounts for 4 data point in the plot. Each point represents the average radius along the vertical direcction and the error bars correspond to the standard deviation. The red line represents the best fit using the snowplow model [1], while the blue line shows its derivative, i.e. the implosion velocity as a function of time. It is observed that during the last stage of the implosion, the velocity increases approximately from $50 km/s$ to $380 km/s$ in $\Delta t \approx50 ns$.

On the other hand, Mach Zehnder interferometry was used to measure the electron density. The interference pattern analysis was done with a combination of Interferometric Data Evaluation Algorithms (IDEA) software [2], [3] and the python pyAbel library to do the inverse Abel transform [4]. Interferometry measurements were made early in time (-100 ns) since the high density and large gradient caused the laser probe to deviate from the collection cone at times close to stagnation. Fig. 2(b) shows the density map obtained using the abovementioned procedure. It shows well-defined edges with a high-density gradient between the interior and the plasma's edge. It can also be seen that the maximum density is around $6\times{10}^{17}{cm}^{-3}$ with an external radius of approximately $8 mm$ and an internal radius of $7 mm$, that is, $\Delta r=1 mm$. These values ​​are used to estimate the density at times close to stagnation. Assuming the same ionization state at -100 ns and -26 ns, and if all electrons are compressed to a column of 3 mm in radius, the density would be $\sim4x{10}^{18}{cm}^{-3}$. While if it is assumed to compress to a radius of 1 mm, the density would be $\sim4x{10}^{19}{cm}^{-3}$. Under these assumptions, the density expected at -26ns is between $1x{10}^{19}{cm}^{-3}$ and $1x{10}^{20}{cm}^{-3}$since ionization is expected to increase when stagnation occurs.


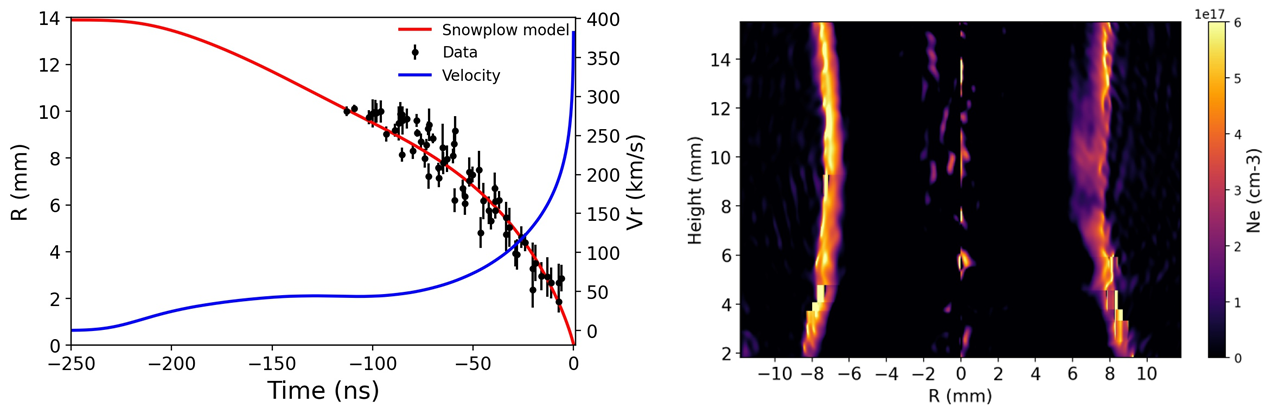


Supplementary figure 2. a) Temporal evolution of pinch radius taken from XUV images Red line represents the best fit using the snowplow model and the velocity in blue is the numerical derivative of the best fit. b) Density map of implosion at -100 ns time obtained by Mach Zehnder interferometer.

**References**

[1] A. Fruchtman, “The snowplow in plasmas of nonuniform density,” *Physics of Fluids B*, vol. 4, no. 4, pp. 855–858, 1992, doi: 10.1063/1.860239.

[2] M. Hippa *et al.*, “Application of Interferometric Fringe Evaluation Software at Technical University Graz.” [Online]. Available: <https://www.optics.tugraz.at/idea/idea.html> .

[3] M. Hipp and P. Reiterer, “User Manual for IDEA 1.7 Software for Interferometrical Data Evaluation,” 2003.

[4] D. D. Hickstein, S. T. Gibson, R. Yurchak, D. D. Das, and M. Ryazanov, “A direct comparison of high-speed methods for the numerical Abel transform,” *Review of Scientific Instruments*, vol. 90, no. 6, Jun. 2019, doi: 10.1063/1.5092635.
